# Newborn Screening Practices and Alpha-Thalassemia Detection — United States, 2016

**DOI:** 10.15585/mmwr.mm6936a7

**Published:** 2020-09-11

**Authors:** M.A. Bender, Careema Yusuf, Tim Davis, M. Christine Dorley, Maria del Pilar Aguinaga, Amanda Ingram, Ming S. Chan, Joseph C. Ubaike, Kathryn Hassell, Jelili Ojodu, Mary Hulihan

**Affiliations:** ^1^Department of Pediatrics, University of Washington, Clinical Research Division, Fred Hutchinson Cancer Research Center, Seattle, Washington; ^2^Association of Public Health Laboratories, Silver Spring, Maryland; ^3^Washington State Newborn Screening, Shoreline, Washington; ^4^Tennessee Department of Health Laboratory Services, Nashville, Tennessee; ^5^Department of Obstetrics and Gynecology and Meharry Sickle Cell Center, Department of Internal Medicine, Nashville, Tennessee; ^6^Tennessee Department of Health Family Health and Wellness; ^7^Florida Department of Health; ^8^Connecticut State Department of Public Health, Katherine A. Kelley State Public Health Laboratory, Rocky Hill, Connecticut; ^9^Division of Hematology, University of Colorado, Aurora, Colorado; ^10^Division of Blood Disorders, National Center on Birth Defects and Developmental Disabilities, CDC.

Alpha-thalassemia comprises a group of inherited disorders in which alpha-hemoglobin chain production is reduced. Depending on the genotype, alpha-thalassemia results in moderate to profound anemia, hemolysis, growth delays, splenomegaly, and increased risk for thromboembolic events; certain patients might require chronic transfusions. Although alpha-thalassemia is not a core condition of the United States Recommended Uniform Screening Panel* for state newborn screening programs, methodologies used by some newborn screening programs to detect sickle cell disease, which is a core panel condition, also detect a quantitative marker of alpha-thalassemia, hemoglobin (Hb) Bart’s, an abnormal type of hemoglobin. The percentage of Hb Bart’s detected correlates with alpha-thalassemia severity. The Association of Public Health Laboratories’ Hemoglobinopathy Workgroup conducted a survey of state newborn screening programs’ alpha-thalassemia screening methodologies and reporting and follow-up practices. Survey findings indicated that 41 of 44 responding programs (93%) report some form of alpha-thalassemia results and 57% used a two-method screening protocol. However, the percentage of Hb Bart’s used for thalassemia classification, the types of alpha-thalassemia reported, and the recipients of this information varied widely. These survey findings highlight the opportunity for newborn screening programs to revisit their policies as they reevaluate their practices in light of the recently released guideline from the Clinical and Laboratory Standards Institute (CLSI) on Newborn Screening for Hemoglobinopathies (*1*). Although deferring to local programs for policies, the report used a cutoff of 25% Hb Bart’s in its decision tree, a value many programs do not use. Standardization of screening and reporting might lead to more timely diagnoses and health care services and improved outcomes for persons with a clinically significant alpha-thalassemia.

Thalassemias are the most common single gene disorders (*2*), with approximately 5% of the world’s population having an alpha-thalassemia variant (*3*). Public health data for the United States are lacking, but in California, 1 in 10,000 newborns has an alpha-thalassemia syndrome (*4*). Prevalence is highest among Laotians and Cambodians and is also found among African, Chinese, Filipino, Mediterranean, Vietnamese, and Thai persons, as well as among those with Middle Eastern ancestry (*3*). Genetic mutations in the alpha-globin gene cluster on chromosome 16 are responsible for alpha-thalassemia, resulting in inefficient production of red blood cells, which affects organ function and growth and results in anemia and iron overload. Most alpha-thalassemias are due to deletion mutations, but there are also less common nondeletion mutations (*5*). Because screening platforms vary in their resolution and sensitivity for detection and quantification of aberrant hemoglobin species, using a different platform for the first round of screening compared with the second round maximizes the number of persons identified with Hb Bart’s levels indicative of alpha-thalassemia.

To better understand newborn screening programs’ alpha-thalassemia screening practices, the Association of Public Health Laboratories’ Hemoglobinopathy Workgroup initiated the first nationwide survey of U.S. newborn screening programs in October 2016. An eight-question survey was e-mailed to all 53 U.S. newborn screening programs. Nonrespondents received reminder e-mails and telephone calls in an effort to maximize the response rate. The e-mail, which was addressed to the main contacts at each newborn screening program (i.e., laboratory directors, laboratory managers, and follow-up staff members), was sent with a survey link, encouraging collaboration to complete one survey per newborn screening program. Questions covered the methods used for testing, number of screening tests used, procedures for reporting of results, and follow-up protocols.

At the end of the survey period, 44 (83%) of the 53 newborn screening programs responded to the survey. All 44 responding programs used methods capable of screening for alpha-thalassemia, and 41 (93%) reported the results. Twenty-five (57%) programs reported use of two modalities for screening for alpha-thalassemia, 14 (32%) reported use of one screening modality only, and two (4.5%) did not provide sufficient information to determine whether they use one or two methods. Among the 25 newborn screening programs that reported using two screening modalities, 15 (60%) used isoelectric focusing (IEF) as their first test, and 10 (40%) used high performance liquid chromatography (HPLC). For a secondary method, 15 (60%) used HPLC, and 10 (40%) used IEF. Among the 14 programs using only one test, eight used IEF, and six used HPLC. The CLSI report does not preferentially recommend a particular method or whether to use one or both technologies.

Patients with more deleted alpha-genes have increased levels of Hb Bart’s and increased clinical severity (Table 1). Each form of alpha-thalassemia is associated with a range of Hb Bart’s, and individual programs determine what thresholds or cutoffs they will use for screening. The Hb Bart’s cutoff percentage used for classifying alpha-thalassemia types varied widely among programs (Table 2), as did the means of reporting of results indicative of alpha-thalassemias. Some reported only that Hb Bart’s was present, some reported a single form of suspected alpha-thalassemia (e.g., Hb H disease) (*3*), and others reported multiple suspected forms (e.g., Hb H disease and alpha-thalassemia trait). Reasons for not reporting elevated Hb Bart’s included the lack of an HPLC setup, inability to confirm or quantify levels, as no Hb Bart’s standard is commercially available, and the absence of alpha-thalassemia on the Recommended Uniform Screening Panel.

**TABLE 1:** Clinical characteristics of different forms of alpha-thalassemia

No. of alpha (α) loci deleted	Genotype (alpha [α] gene configuration)	Classification	Clinical features	Hb and RBC indices*^,†^		% Hb Bart’s in newborns^§^ (values vary depending on testing method)
0	(αα/αα)	Normal	Normal	Hb:	Male: 5.9 ± 1.0 g/dL	None
					Female: 14.0 ± 0.9 g/dL	
				MCV:	Male: 89.1 ± 5.01 fl	
					Female: 87.6 ± 5.5 fl	
				MCH:	Male: 30.9 ± 1.91 fl	
					Female: 30.2 ± 2.1 fl	
1	(α–/αα)	Silent alpha-thalassemia carrier: alpha-thalassemia 2 heterozygote	None	Hb:	Male: 14.3 ± 1.4 g/dL	1–3
					Female: 12.6 ± 1.2 g/dL	
				MCV:	81.2 ± 6.9 fl	
				MCH:	26.2 ± 2.3 pg/cell	
2	(α–/α–) or (–/αα)	Alpha-thalassemia trait: alpha-thalassemia 2 homozygote or alpha-thalassemia 1 heterozygote	Mild anemia, microcytosis	Hb:	Male: 13.9 ± 1.7 g/dL	3–6
					Female: 12.0 ± 1.0 g/dL	
				MCV	71.6 ± 4.1 fl	
				MCH	22.9 ± 1.3 pg/cell	
3	(α–/–)	Hb H disease / alpha-thalassemia intermedia	Moderate to severe anemia	Hb:	Male: 10.9 ± 1.0 g/dL	5–30
					Female: 9.5 ± 0.8 g/dL	
				MCV:	Children: 56 ± 5 fL	
					Adults: 61 ± 4 fl	
				MCH:	18.4 ± 1.2 pg/cell	
4	(–/–)	Homozygous alpha-thalassemia / alpha-thalassemia major/Bart’s hydrops fetalis	Fetal death with hydrops fetalis	Hb:	3–8 g/dL	100
				MCV:	136 ± 5 fL	
				MCH:	31.9 ± 9 pg/cell	

**Abbreviations:** fl = femtoliter (10−^15^ liter); Hb = hemoglobin; Hb H = hemoglobin H; MCH = mean corpuscular hemoglobin; MCV = mean corpuscular volume; pg = picogram (10−^12^ gram); RBC = red blood cell.

* Higgs DR, Bowden DK. Clinical and laboratory features of the alpha-thalassemia syndromes. In: Steinberg MH, Forget PG, Higgs DR, Nagel RL, eds. Disorders of hemoglobin: genetics, pathophysiology, and clinical management. Cambridge, United Kingdom: Cambridge University Press; 2001:431–69.

^†^ Origa R, Moi P. Alpha-thalassemia. In: Adam MP, Ardinger HH, Pagon RA, et al., eds. GeneReviews. Seattle, WA: University of Washington; 1993–2020.

^§^ Hb Bart’s is not present after age 1 year.

**TABLE 2:** Reporting and recipients of alpha-thalassemia screening results — 41 newborn screening programs, United States, 2016

Characteristic	Alpha-thalassemia type				
	Alpha-thalassemia major	Hb H disease	Alpha-thalassemia trait	Silent alpha-thalassemia carrier	Other (i.e., unspecified Bart’s)
**No. (%) of programs reporting results**	15 (37)	20 (49)	20 (49)	7 (17)	22 (54)
Recipient of results*					
Physician	9	14	14	5	13
Parent	2	3	3	1	1
NBS follow-up	11	16	16	5	16
Other	1	2	2	1	12
Unknown	3	3	2	1	1
**No. (%) of programs that provided percentage cutoffs of Hb Bart’s for reporting results**	7 (17)	6 (15)	9 (22)	3 (7)	9 (22)
**Cutoff percentage of Hb Bart’s for reporting results**					
Average	64	21.5	9.1	7	15
Minimum	25	11	2	3	3
Maximum	100	31	20	11	25

**Abbreviation:** Hb H = hemoglobin H; NBS = newborn screening.

* Categories are not mutually exclusive; newborn screening programs report multiple results.

Programs that report results indicative of alpha-thalassemia disseminate the results differently. The majority of laboratories report to newborn screening follow-up programs, which are responsible for disseminating screening results and recommendations for confirmatory testing, and to the provider; parents are less likely to receive direct notification. Other recipients of screening results include birthing hospitals, hematologists, regional sickle cell specialty centers, and contracted specialists. Overall, 33 (80%) of 41 newborn screening programs that report results provided recommendations for patient retesting or follow-up. These recommendations were largely dependent on the percentage of Hb Bart’s; recommendations included confirmatory testing, complete blood count, reticulocyte count, genetic counseling, and referral to a pediatric hematologist.

## Discussion

This report describes the first nationwide survey to determine whether newborn screening programs report alpha-thalassemia screening results and how they report the findings on phenotype/genotype and follow-up practices to health care providers and parents. Overall, >90% of responding programs report some level of elevated Hb Bart’s. As a result, the newborns identified with a form of alpha-thalassemia by these programs might be able to access specialty medical care at a young age, if needed. Nonetheless, the findings of this analysis also reveal considerable program-to-program variability in 1) the screening platforms used, 2) the process or cut-offs used to define specific forms of potential alpha-thalassemia, 3) the types of alpha-thalassemia reported, and 4) how and to whom information is reported.

A concerning finding is that 20% of programs that report results indicative of alpha-thalassemia do not make recommendations for follow-up; this suggests a potential opportunity for further research to determine whether standardization across programs might lead to improved health outcomes. The finding that few of the newborn screening programs notify parents about positive alpha-thalassemia results is not unique to this condition. Similar findings have been reported regarding notification of parents of newborn screening results indicating sickle cell disease and sickle cell trait (*6*). However, this practice suggests another possible area for study to determine whether early parental knowledge and education might result in more timely initiation of care for affected children.

The potential impact of working to standardize newborn screening for alpha-thalassemia extends far beyond the identification during infancy of those with disease states, as well as those who are carriers. It also suggests an opportunity to collect data that could better define the birth incidence and spectrum of this condition in the United States. Clinically, newborn screening for elevated Hb Bart’s allows those with Hb H disease to receive appropriate referrals to hematologists and thereby avoid complications of untreated disease. Newborn screening could also reduce the risk for those with alpha-thalassemia trait, who might receive a misdiagnosis of iron deficiency, from receiving inappropriate courses of iron therapy as well as delays in receipt of a definitive diagnosis. Early identification also provides the opportunity for genetic counseling and education with a focus on identifying mothers at risk for a hydrops fetalis pregnancy and risk to maternal health from a stillbirth, in addition to the risk to the fetus’s life. This is becoming increasingly important as interventions to rescue, and even cure, such pregnancies in utero are improving (*7*). Newborn screening for alpha-thalassemia provides an opportunity for the education of affected families and their health care providers about this condition as part of the follow-up component of the newborn screening program.

The findings in this report are subject to at least one limitation. Nine programs (17%) did not respond to the survey, although it was determined that four of the nonrespondents are known to send specimens to programs that did respond to the survey. As such, this survey represents information from newborn screening programs that cover 86% of births in the United States.

The infrastructure for universal newborn screening and reporting of alpha-thalassemia in the United States already exists, and there are many opportunities for standardizing and streamlining the process. The results of this survey could guide further discussion, development of definitions, and dissemination of evidence-based best practices and expert guidelines for improving upon this work^†^^,^^§^ (*1*,*8*). As the demographics of the U.S. population change to include more persons from areas where alpha-thalassemia is prevalent (e.g., Southeast Asia, China, and the Middle East) (*3*), the number of U.S. residents with a form of alpha-thalassemia might also increase. Uniformity of screening, diagnosis, and treatment for alpha-thalassemia could play an important role in increasing timely and appropriate health care. An increase in the number of newborn screening programs reporting alpha-thalassemia results for multiple suspected forms of the condition might 1) improve access to specialty care before the occurrence of severe complications, 2) increase genetic counseling, and 3) provide data needed to better understand the public health impact and clinical outcomes of alpha-thalassemia in the United States. Meanwhile, efforts continue toward developing definitions for uniform minimum capabilities for testing and uniform suggested follow-up.

**Summary**

**What is already known about this topic?**

Despite a 5% global prevalence, alpha-thalassemia is not a core condition on the United States Recommended Uniform Screening Panel for state newborn screening (NBS) programs. However, NBS methodologies used to detect sickle cell disease, reported by all states, also detect alpha-thalassemia.

**What is added by this report?**

A 2016 survey of NBS programs found that although most programs report at least one form of suspected alpha-thalassemia, the methodologies, thresholds used, forms of disease reported, and processes for reporting vary widely.

**What are the implications for public health practice?**

Standardization of technical and reporting procedures could provide data to better understand the public health impact and clinical outcomes of alpha-thalassemia, ensure appropriate health care, and improve outcomes.

## Footnotes

*
https://www.hrsa.gov/advisory-committees/heritable-disorders/rusp/index.html .

† https://www.aphl.org/programs/newborn_screening/Pages/Hemoglobinopathies.aspx.

§ https://thalassemia.com/documents/SOCGuidelines2012.pdf.
